# Pigmented Nodular Basal Cell Carcinomas in Differential Diagnosis with Nodular Melanomas: Confocal Microscopy as a Reliable Tool for *In Vivo* Histologic Diagnosis

**DOI:** 10.1155/2011/406859

**Published:** 2010-10-14

**Authors:** Alice Casari, Giovanni Pellacani, Stefania Seidenari, Anna Maria Cesinaro, Francesca Beretti, Patrizia Pepe, Caterina Longo

**Affiliations:** ^1^Department of Dermatology and Venereology, University of Modena and Reggio Emilia, Via del Pozzo 71, 41100 Modena, Italy; ^2^Department of Pathology, University of Modena and Reggio Emilia, Via del Pozzo 71, 41100 Modena, Italy; ^3^Department of Anatomy and Histology, University of Modena and Reggio Emilia, Via del Pozzo 71, 41100 Modena, Italy

## Abstract

Nodular basal cell carcinoma, especially when pigmented, can be in differential diagnosis with nodular melanomas, clinically and dermoscopically. Reflectance confocal microscopy is a relatively new imaging technique that permits to evaluate in vivo skin tumors with a nearly histological resolution. Here, we present four cases of challenging nodular lesions where confocal microscopy was able to clarify the diagnosis.

## 1. Introduction

Nodular lesions continue to pose a diagnostic challenge to even the most experienced physicians. Among nodular lesions, nodular basal cell carcinoma (BCC), especially in its pigmented variant, is one of the most difficult-to-diagnose lesions. The differential diagnosis, between BCC and other skin cancers, is of critical importance for the consequences and serious morbidity of an undiagnosed tumor. Because of its relative rapid growth with bleeding as a common symptom, invasive nodular melanoma (NM) is the main differential diagnosis of pigmented BCC. Clinically, pigmented BCC shares the features of the corresponding histological subtype of its nonpigmented variant. It appears as a sharply demarcated nodule with a long-standing history of growth and often showing ulceration. Conversely, NM usually starts as an expanding papule that increases in size quite rapidly. Both kinds of nodules, BCC and NM, cannot be distinguished by clinical examination alone. In fact, the ABCD criteria do not apply to the tumor nodule itself, which is commonly symmetric with smooth borders. Additionally, the color is often quite homogeneous, ranging from black, bluish, to dark brown, and sometimes the surface of the tumor is shiny or ulcerated with bleeding. Diameter is usually small and it can be less than 6 mm. For all these reasons, clinical diagnosis of nodules may be subtle and misdiagnosis is not infrequent [1].

Dermoscopy is a noninvasively method that has been reported to be a useful tool for the early and accurate recognition of pigmented lesions of the skin [2, 3]. Its use increases diagnostic accuracy between 5 and 30% over clinical visual inspection, depending on the type of skin lesion and the experience of the physician [4]. However, nodular lesions can lack specific dermoscopic criteria being completely or partially featureless in their appearance.

Reflectance confocal microscopy (RCM) in an emerging noninvasive diagnostic tool that provides *in vivo* tissue images at nearly cellular histological resolution. RCM employs a low-power laser beam (near-infrared wavelength) that scans the skin horizontally, producing highly detailed black and white images from the epidermis to the upper papillary dermis with an imaging depth of up to 200–300 *μ*m. The RCM holds the unique opportunity to non invasively assess cellular and architectural aspects of skin lesions usually seen with conventional histopathology. Images are easily obtained without producing any sort of artifacts of the examined skin areas. Furthermore, skin lesions can be “histologically” examined over time and followed up. Evaluation of skin tumors is routinely conducted by the examination of mosaics and/or series of consecutive high-resolution images from the epidermis up to superficial dermis. Characteristic confocal findings for pigmented skin lesions, including BCCs and MM, have been reported. The striking confocal features for MM diagnosis include the presence of nonedged papillae corresponding to a disarray of the rete ridge, cytological atypia at the dermal-epidermal junction, the presence of round pagetoid cells, the presence of nucleated cells corresponding to melanocytes infiltrating the dermal papillae, and the cerebriform nests in the dermis correlated to the presence of large aggregates of small melanocytes [5, 6]. BCC diagnosis is usually made by analyzing the lesion for the presence of five relevant RCM criteria which include the presence of elongated monomorphic basaloid nuclei, polarization of these nuclei along the same axis of orientation, prominent inflammatory infiltrate, increased dermal vasculature, and pleomorphism of the overlying epidermis indicative of actinic damage [7–9]. Another relevant criterion for BCC diagnosis is the presence of tightly packed cells arranged to form basaloid nests [10].

Here, we describe four patients with clinically and dermoscopically equivocal nodular lesions for which RCM examination allowed for a rapid and accurate prebiopsy diagnosis.

## 2. Materials and Methods

Dermoscopic images were obtained by using a videodermoscope (FotoFinder, Teachscreen, Germany). Confocal imaging was performed with near-infrared reflectance-mode confocal laser scanning microscope (Vivascope1500; Lucid Inc, Rochester, New York). The instrument uses a diode laser at 830 nm with a power of less than 16 mW at tissue level and ×30 water-immersion lens enabling a horizontal optical resolution of 2 *μ*m and a vertical resolution of 5 *μ*m. Instruments and acquisition methods have been described elsewhere [11]. Each individual RCM image (0.5 × 0.5 mm) corresponds to a horizontal section, at a selected tissue depth, with a lateral resolution of 1.0 *μ*m and an axial resolution of 3 to 5 *μ*m. A sequence of full-resolution individual images at a given depth were acquired for each lesion and “stitched” together to create a mosaic area with an area between 4 × 4 and 8 × 8 mm. Images resolution was of 2 pixels/*μ*m, generating 8000 × 8000-pixel and 16000 × 16000-pixel images, respectively. A minimum of 3 mosaics was obtained per lesion at three different skin levels: superficial epidermal layers (stratum granulosum-spinosum), dermo-epidermal junction, and papillary dermis. Spatial orientation of lesions for dermoscopy and confocal correlation was performed using an external macrocamera (ViVaCam; Lucid Inc.) adapted to the confocal microscope.

After tumor excision, the tissue was fixed in formalin and embedded in paraffin. After routine processing, the slides were stained with hematoxylin-eosin. Digital photographs of histologic sections were obtained by means of a white light microscope (Nikon eclipse 80i, Nikon, Japan) equipped with a Nikon's Digital Sight DS-L2.

## 3. Results

### 3.1. Case 1

A 58-year-old man referred to our clinic for the presence of a pigmented nodular lesion on his left temple. Clinically, the lesion was a 6 mm sharply demarcated pigmented nodule with a blue hue.

**Figure 1:**
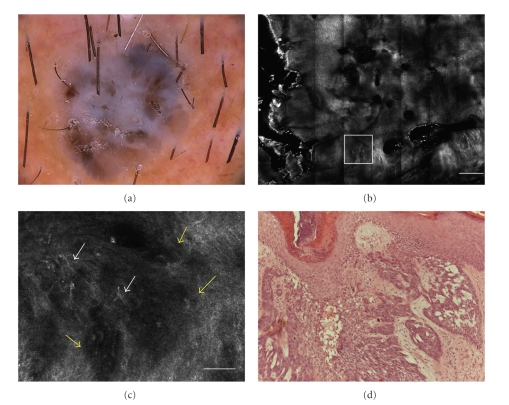
(a) Dermoscopic image of nodular pigmented lesion showing a blue gray veil structureless brown areas and dark brown dots in the center (original magnification 30×). (b) RCM image (4 × 4 mm) at dermoepidermal level, showing a general cauliflower architecture highly specific for BCC diagnosis (scale bar: 500 *μ*m). (c) RCM image (0.5 × 0.5 mm, white frame), showing dark silhouettes (*yellow arrows*) outlined by dark spaces. In the inner portion of them, bright filaments were present (*white arrows*) (scale bar: 50 *μ*m). (d) Histology showing basaloid nodules in the dermis corresponding to hypopigmented dark silhouettes. Within and outside the tumor nests some melanin was present (H&E section; original magnification ×100).

Dermoscopy showed blue-white veil, irregularly distributed black-brown dots, and brown structureless areas (Figure 1(a)). No clear-cut diagnostic dermoscopic features were present.

RCM mosaic image showed a cauliflower architecture due to solid units of tumor cells showing up as hypo-reflective aggregates (dark silhouette) surrounded by dark areas (Figure 1(b)).

At high resolution, dark silhouettes appeared as hypo-reflective shadows outlined by a dark cleft, with a well correspondence to basaloid islands surrounded by dense stromal collagen. In the inner portion of the dark silhouette, it was possible to note the hyperreflective thin dendrites and bright oval structures corresponding to pigmented melanocytes or inflammatory cells on histology (Figure 1(c)).

Upon histology dark silhouettes corresponded to basaloid nodules in the dermis; within and outside the tumor nests melanin was present as dendritic melanocytes, clumps of free melanin, or melanin inside melanophages (Figure 1(d)).

### 3.2. Case 2

A 73-year-old man presented a nodular pigmented lesion on the back with unknown history of changing lesion or fast growth. 

Clinically the lesion appeared as a 8 mm nodule, heavily pigmented, irregular in shape, with indented borders.

**Figure 2:**
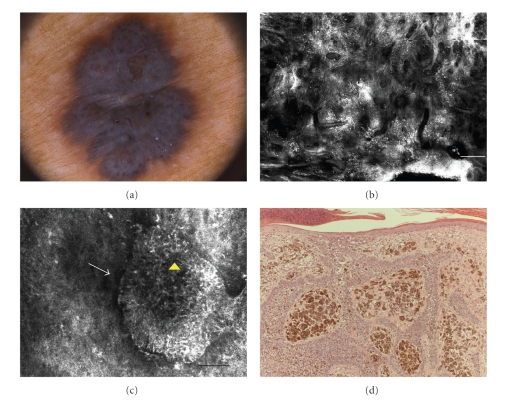
(a) Dermoscopic image of pigmented nodule exhibiting a bluish veil on the entire lesion and numerous vessels with corkscrew aspect (original magnification 20×). (b) RCM image (4 × 4 mm) imaged at upper dermis level, showing the cauliflower aspect with numerous plump bright cells overlying all lesion area (scale bar: 500 *μ*m). (c) RCM image (0.5 × 0.5 mm) showing reflective aggregates of tightly packed cells with peripheral palisading (*white arrow*) surrounded by dark clefts. Inside the tumoral nest bright filaments and dots were present (*yellow arrowhead*) (scale bar: 50 *μ*m). (d) Histology showing basaloid tumoral nests enclosed by connective tissue stroma. Melanin was present inside the tumoral nests and also in the surrounding stroma where numerous melanophages were detected (H&E section; original magnification ×100).

On dermoscopy, this lesion was relatively featureless, revealing a bluish veil overlying the entire lesion, and several vessels with corkscrew aspect (Figure 2(a)).

RCM examination at low magnification (mosaic) at DEJ exhibited a cauliflower global architecture and a large amount of enlarged vessels. Higher magnification (0.5 × 0.5 mm) revealed numerous reflective aggregates of tightly packed cells with peripheral palisading surrounded by dark clefts. Inside the tumoral nest bright filaments and dots were present (Figure 2(c)).

These findings were suggestive of BCC, and this lesion was removed by excisional biopsy. Histopathologic exam demonstrates nodular masses of basaloid cells extending to dermis and enclosed by connective tissue stroma arranged in bundles. Melanin pigment was present either as melanophages or melanocytes entrapped in the basaloid nests (Figure 2(d)).

### 3.3. Case 3

**Figure 3:**
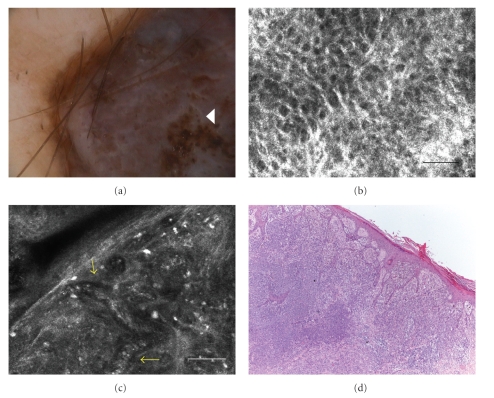
(a) Dermoscopic image of nodular pigmented lesion with an atypical globular pattern, structureless dark brown areas, irregular brown dots in the center of the lesion (*white arrowhead*), and a blue-white veil (original magnification 50×). (b) RCM image (0.5 × 0.5 mm) acquired at stratum granulosum-spinosum, showing broadened honeycombed (scale bar: 50 *μ*m). (c) RCM image (0.5 × 0.5 mm), at dermal level, showing markedly pleomorphic cells distributed in sheet-like structures in the dermis and numerous bright dots. Inside the tumoral nested proliferation, new-born vessels are present (*yellow arrows*) (scale bar: 50 *μ*m). (d) Histological image showing an overview of the nodular melanoma with Breslow thickness of 6.5 mm (H&E section; original magnification ×400).

A 44-year-old man was concerned for the presence of a nodular pigmented lesion on his right forearm. Clinically the lesion was a 1,5 cm dark brown symmetric nodule with sharp borders. Dermoscopy showed an atypical globular pattern, structureless dark brown areas, irregular brown dots in the center of the lesion, and a blue-white veil (Figure 3(a)).

RCM image revealed a thin epidermis with broadened-honeycombed pattern, consisting of large polygonal cells and thickened bright border (Figure 3(b)).

At the DEJ, single nucleated cells or atypical melanocytes tend to infiltrate the dermal papillae and disarrange the architecture. Markedly pleomorphic cells with bright cytoplasm and dark nuclei are distributed in sheet-like structures in the dermis, numerous bright dots representing inflammatory cells overlying all lesion and enlarged vessels (Figure 3(c)). The RCM findings were highly suggestive for melanoma diagnosis.

Histologic sections showed tumoral nests of malignant melanocytes in the upper dermis, irregular dense clusters, and atypical nucleated cells in the upper dermis. The lesion was a melanoma with a Breslow thickness of 6.5 mm (Figure 3(d)).

### 3.4. Case 4

A 60-year-old man referred to our clinic for the presence of a pigmented nodular lesion on the back. Clinically it was a 1 cm lesion with sharp borders, asymmetrical in shape, dark brown to blue in color, presenting a large area of ulceration in the center of the nodule.

**Figure 4:**
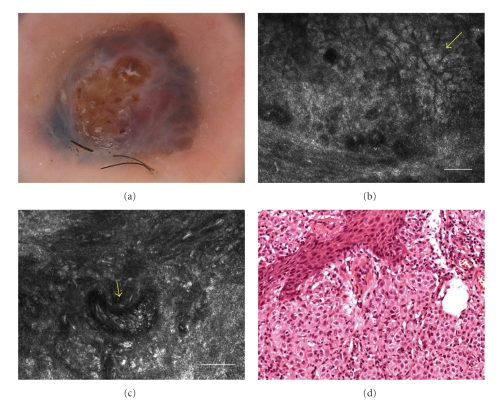
(a) Dermoscopy showed structureless areas with blue hue and a globular pattern. Ulceration was present in the center of the nodule (original magnification 30×). (b) RCM image (0.5 × 0.5 mm) at dermal level, showing pleomorphic small cells (*yellow arrow*) arranged to form a cerebriform nests delimitated by fibrous septa (scale bar: 50 *μ*m). (c) RCM image (0.5 × 0.5 mm) at dermal level, showing an enlarged new-born vessel with a prominent blood flow (*yellow arrow*) (scale bar: 50 *μ*m). (d) Histology showing a solid proliferation of atypical melanocytes in which new-born vessels can be recognized (H&E section; original magnification ×100).

Dermoscopy showed structureless areas with blue hue and a globular pattern. Ulceration was present in the center of the nodule (Figure 4(a)).

Upon RCM, at epidermal level, the lesion exhibited very few large pagetoid cells. The DEJ was not visible for the presence of a marked architectural disarrangement of the rete ridge. Numerous atypical cells with the tendency to form aggregates with cerebriform aspect were present in the dermis (Figure 4(b)). The lesion showed a marked and increased vascularization with enlarged new-born vessels (Figure 4(c)).

Histology revealed an NM of 2.06 mm Breslow thickness characterized by fully transformed melanocytes forming a solid proliferation into the dermis (Figure 4(d)).

## 4. Discussion

The clinical and dermatoscopic examination is very useful in evaluating and diagnosing pigmented skin lesions [12–15]. For nodular lesions, the correct diagnosis may be very difficult in many cases especially when dealing with tumors lacking dermatoscopic clues specific for melanocytic or not melanocytic entities. In fact, despite careful and meticulous clinical inspection and dermatoscopic evaluation, nodular lesions often remain diagnostically challenging. Confocal microscopy represents a useful adjunct to dermoscopy in the hands of physicians while allowing an accurate morphological analysis of the lesion at histologic resolution. 

The current series of patients from our clinic demonstrates the potential use of RCM in the evaluation of nodules that are diagnostically equivocal both clinically and dermoscopically. All cases showed a bluish hue and structureless areas. In dermoscopy the perception of blue pigmentation represents a relevant clue for MM diagnosis: the identification of a blue hue within a lesion represents a good predictor of malignancy, but not a specific one. To improve the diagnostic efficacy of the blue color, the dermoscopic distinction between a bluish diffuse or speckled pigmentation (the blue areas) and a dark blue pigmentation underlying a whitish veil (the blue-whitish veil) was suggested [16, 17] but showed a poor intra- and interobserver reproducibility [2].

RCM enabled *in vivo* identification of characteristic cytological and architectural substrates underlying the blue hue in dermoscopy: it permits the distinction of blue areas from blue-whitish veil. The former is characterized by plump cells with ill-defined borders corresponding to melanophages and inflammatory infiltrate upon histology, the latter by the contemporary presence of epidermal and dermal features consistent with diagnosis of melanoma, such as disarranged pattern, pagetoid cells, cytologic and architectural atypia, nonhomogeneous and cerebriform clusters, and dermal nucleated cells [18].

In our study, dermoscopic evaluation revealed an aspecific and not clear-cut features for diagnosing the nodule as being melanocytic or nonmelanocytic in nature. Altamura et al. showed that the frequency of melanocytic patterns linearly increased with the pigmentation of the lesions, having a prevalent distribution in the heavily pigmented BCCs. This means that, among pigmented BCCs, the heavily pigmented variant, like in our cases, represents the most difficult type to be differentiated from melanocytic lesions [12]. 

González et al. first described five relevant criteria for the diagnosis of BCC by RCM, which was later validated in a larger study [7, 19]. Later on, further descriptors were added, such as the presence of tightly packed cells forming basaloid nests outlined by a dark cleft [20]. Additionally, the presence of dendritic cells in BCC nests was correlated with melanocytes whereas dendritic cells in the epidermis corresponded to Langerhans cells [9]. Melanocytes were found typically in pigmented BCCs where they appear as long dendritic shaped cells entrapped inside the tumor islands. Along with melanocytes, the pigmentation upon RCM was related to the presence of inflammatory infiltrate showing up as bright spots or to ill-defined plump bright cells corresponding to melanin-rich melanophages.

RCM has been demonstrated to be crucial in discriminating the nature of the four nodules. On one hand, BCC lesions were characterized by the presence of a general cauliflower aspect which could be due to dark silhouettes, poor in pigmentation, or reflective aggregates of tightly packed cells with peripheral palisading in which the pigment was more prominent. The blue color is due to the dermal location of the basaloid nests and, also, to the bright filaments and dots present within and around tumoral nests, which correspond to inflammatory cells and melanophages upon histology.

On the other hand, the blue hue in NM was due to the chaotic and disarranged dermal compartment with prominent atypical cellularity and to the epidermal thinning. In fact, the presence of cerebriform nests and sheet-like structure represents highly specific diagnostic criteria for invasive melanoma [20, 21]. Although confocal microscopy has a limited laser depth penetration allowing to carefully explore the upper portion of the papillary dermis, in our cases, RCM diagnosis was possible because both BCCs and NM are always characterized by having a thinning and eroded epidermis. This makes it possible to detect the most striking features of both entities even the dermal ones.

Our cases demonstrate the use of RCM in clinical practice in the evaluation of pigmented nodules that are diagnostically equivocal both clinically and dermoscopically. The RCM-based diagnosis was made within about five minutes of RCM scanning allowing to correctly judge the lesion and choose the most appropriate clinical management. However, it has to be taken into account that a confident RCM diagnosis is always operator dependent and a large experience and specific training in confocal microscopy is warranted.

## References

[1] Menzies SW, Westerhoff K, Rabinovitz H, Kopf AW, McCarthy WH, Katz B (2000). Surface microscopy of pigmented basal cell carcinoma. *Archives of Dermatology*.

[2] Argenziano G, Soyer HP, Chimenti S (2003). Dermoscopy of pigmented skin lesions: results of a consensus meeting via the internet. *Journal of the American Academy of Dermatology*.

[3] Kittler H, Pehamberger H, Wolff K, Binder M (2002). Diagnostic accuracy of dermoscopy. *Lancet Oncology*.

[4] Carli P (2007). Dermoscopy not yet shown to increase sensitivity of melanoma diagnosis in real practice. *Archives of Dermatology*.

[5] Guitera P, Pellacani G, Crotty KA (2010). The impact of in vivo reflectance confocal microscopy on the diagnostic accuracy of lentigo maligna and equivocal pigmented and nonpigmented macules of the face. *Journal of Investigative Dermatology*.

[6] Guitera P, Pellacani G, Longo C, Seidenari S, Avramidis M, Menzies SW (2008). In vivo reflectance confocal microscopy enhances secondary evaluation of melanocytic lesions. *Journal of Investigative Dermatology*.

[7] Nori S, Rius-Díaz F, Cuevas J (2004). Sensitivity and specificity of reflectance-mode confocal microscopy for in vivo diagnosis of basal cell carcinoma: a multicenter study. *Journal of the American Academy of Dermatology*.

[8] Ahlgrimm-Siess V, Hofmann-Wellenhof R, Cao T, Oliviero M, Scope A, Rabinovitz HS (2009). Reflectance confocal microscopy in the daily practice. *Seminars in Cutaneous Medicine and Surgery*.

[9] Segura S, Puig S, Carrera C, Palou J, Malvehy J (2007). Dendritic cells in pigmented basal cell carcinoma: a relevant finding by reflectance-mode confocal microscopy. *Archives of Dermatology*.

[10] Agero AL, Busam KJ, Benvenuto-Andrade C (2006). Reflectance confocal microscopy of pigmented basal cell carcinoma. *Journal of the American Academy of Dermatology*.

[11] Scope A, Benvenuto-Andrade C, Agero A-LC (2007). In vivo reflectance confocal microscopy imaging of melanocytic skin lesions: consensus terminology glossary and illustrative images. *Journal of the American Academy of Dermatology*.

[12] Altamura D, Menzies SW, Argenziano G (2010). Dermatoscopy of basal cell carcinoma: morphologic variability of global and local features and accuracy of diagnosis. *Journal of the American Academy of Dermatology*.

[13] Argenziano G, Ferrara G, Francione S, Nola KD, Martino A, Zalaudek I (2009). Dermoscopy—the ultimate tool for melanoma diagnosis. *Seminars in Cutaneous Medicine and Surgery*.

[14] Scalvenzi M, Lembo S, Francia MG, Balato A (2008). Dermoscopic patterns of superficial basal cell carcinoma. *International Journal of Dermatology*.

[15] Segura S, Pellacani G, Puig S (2008). In vivo microscopic features of nodular melanomas: dermoscopy, confocal microscopy, and histopathologic correlates. *Archives of Dermatology*.

[16] De Giorgi V, Massi D, Trez E, Salvini C, Quercioli E, Carli P (2003). Blue hue in the dermoscopy setting: homogeneous blue pigmentation, gray-blue area, and/or whitish blue veil?. *Dermatologic Surgery*.

[17] Pellacani G, Bassoli S, Longo C, Cesinaro AM, Seidenari S (2007). Diving into the blue: in vivo microscopic characterization of the dermoscopic blue hue. *Journal of the American Academy of Dermatology*.

[18] Seidenari S, Ferrari C, Borsari S (2010). Reticular grey-blue areas of regression as a dermoscopic marker of melanoma in situ. *British Journal of Dermatology*.

[19] González S, Tannous Z (2002). Real-time, in vivo confocal reflectance microscopy of basal cell carcinoma. *Journal of the American Academy of Dermatology*.

[20] Pellacani G, Cesinaro AM, Seidenari S (2005). Reflectance-mode confocal microscopy of pigmented skin lesions—improvement in melanoma diagnostic specificity. *Journal of the American Academy of Dermatology*.

[21] Pellacani G, Cesinaro AM, Seidenari S (2005). In vivo confocal reflectance microscopy for the characterization of melanocytic nests and correlation with dermoscopy and histology. *British Journal of Dermatology*.

